# Assessment of validity, reliability, and feasibility of OMERACT ultrasound knee osteoarthritis scores in Egyptian patients with primary knee osteoarthritis

**DOI:** 10.1007/s10067-024-07171-4

**Published:** 2024-10-18

**Authors:** Manal Abd El Moniem El Menyawi, Galila Gamal, Hoda Abdelbadie, Rasmia Elgohary

**Affiliations:** 1 Rheumatology and Clinical Immunology Subspecialty, Internal Medicine Department, Kasr Alainy School of Medicine, Cairo University, Cairo University Hospitals, Al-Saray St., El-Maniel, Cairo, 11562 Egypt; 2 Rheumatology and Clinical Immunology Subspecialty, Internal Medicine Department, Fayoum University, Fayoum, Egypt

**Keywords:** Knee osteoarthritis, OMERACT score, Reliability, Ultrasound, Validity

## Abstract

**Background:**

Ultrasound (US) can evaluate all joint components affected by knee osteoarthritis (KOA); however, standardized scoring of US-detected pathology is needed to improve its diagnostic and monitoring capabilities.

**Objectives:**

To examine the validity, reliability, and feasibility of the Outcome Measures in Rheumatology (OMERACT) ultrasound scoring for KOA, comparing with clinical and radiography measures, using predefined cutoff values.

**Methods:**

This cross-sectional study included 75 Egyptian patients with primary KOA. All patients had Western Ontario and McMaster Universities Osteoarthritis Index (WOMAC) score, bilateral knee radiography, and ultrasonography. Inter-observer reliability of ultrasound was evaluated in 30 knees by another newly trained operator.

**Results:**

Most of the OMERACT-US KOA scores showed significant associations with WOMAC clinical scores, except for femoral cartilage damage and effusion. The synovitis score was significantly associated with WOMAC–pain score (*p*-value 0.046), while medial meniscus extrusion (MME) and medial osteophytes were significantly associated with WOMAC–stiffness score (*p*-value 0.009 and 0.023, respectively). MME and synovitis were significantly associated with WOMAC–physical score (*p*-value 0.035 and 0.020, respectively). The ultrasound scores also showed a strong correlation with radiographic scoring. Inter-observer reliability ranged from moderate to excellent agreement (*k* = 0.58 to *k* = 0.83); it was highest for lateral osteophytes (*k* = 0.83), good agreement for synovitis (*k* = 0.72), any osteophytes (*k* = 0.71), damage of femoral cartilage (*k* = 0.70), and moderate agreement for medial osteophytes (*k* = 0.58) and MME (*k* = 0.59).

**Conclusion:**

OMERACT-US scoring system for KOA demonstrated validity, reliability, and feasibility for evaluating both structural and inflammatory components. Using cutoff values improved the scoring reliability for osteophytes and MME.

**Table:** 

**Key Points** *• OMERACT-US scores provide a valid assessment of inflammatory and structural components of knee osteoarthritis.* *• The following changes may improve the performance of the OMERACT-US scores.* a. *The binary score for effusion and synovial hypertrophy can be omitted, as they have no added value.* b. *A semi-quantitative grading for effusion may capture the impact of effusion on clinical outcomes.* c. *Added cutoff values to score medial meniscal extrusion, osteophytes, and pathological effusion improved the respective scores’ reliability.* d. *Applying the updated OMERACT definition of synovitis.* *• OMERACT-US scores are reliable to be used with a newly trained operator, particularly when cutoff values are included, and proper training time is provided.* *• The OMERACT-US score is feasible to be used in clinical practice, as the time taken to perform was short, even for a newly trained operator.*

**Supplementary Information:**

The online version contains supplementary material available at 10.1007/s10067-024-07171-4.

## Introduction

Knee osteoarthritis (KOA) is a common disabling disease characterized by progressive cartilage injury with subsequent structural and inflammatory changes in all joint components, resulting in pain, stiffness, and functional limitations [1]. While conventional radiography remains the mainstay imaging for the assessment of KOA, it lacks sensitivity in early stages and cannot detect inflammatory abnormalities [2]. Although MRI is highly sensitive, its expense and difficulty for serial follow-up assessments limit its use in routine clinical practice [3]. Ultrasonography (US) has gained widespread use in the musculoskeletal field due to its unique advantages; it involves no ionizing radiation, can be repeated as needed, is widely available, easy to use, and can visualize soft tissue structures [4]. The development of ultrasound as a tool for assessing KOA has seen significant milestones over the past decades. Early studies explored the potential of ultrasound in visualizing joint structures affected by KOA [5]. A breakthrough came with the EULAR report on the validity of ultrasound in detecting synovial inflammation and effusion in painful knee OA and its utility in clinical decisions [6, 7]. The formation of the Outcome Measures in Rheumatology (OMERACT) Ultrasound Task Force in 2004 marked a crucial step towards developing consensus definitions for pathological features, which were updated in 2019 [8, 9]. A systematic review and meta-analysis by Oo et al. provided evidence for the validity and reliability of the ultrasound-detected pathologies in KOA. They identified good reliability and validity for most features, particularly with quantitative assessments, while noting variability in definitions and scoring systems. Ultrasound performance varies depending on the specific feature evaluated and the comparison method [10]. Efforts have also focused on developing grading systems for individual pathologies and comprehensive scoring systems [11–19].

In 2014, the OMERACT musculoskeletal ultrasonography (MSUS) group used international consensus and reliability testing to develop standardized knee ultrasound scanning methods and grading scores for both structural and inflammatory abnormalities of KOA. The scores were based on binary and semi-quantitative grading. Although conducted by experts, the inter-observer reliability was fair for synovial effusion, synovial hypertrophy, and cartilage damage and moderate for meniscal extrusion and osteophytes [20]. These scores have been validated against pain severity, radiography, and MRI [21].

Despite these advancements, several areas remain underexplored, including limited validation studies in diverse ethnic populations, particularly from African countries; insufficient data on the correlation between OMERACT-ultrasound scores and patient-reported outcomes across diverse cultures and healthcare systems; variability in equipment and operator skills; and finally a few studies investigating the feasibility of implementing the standardized OMERACT-ultrasound scoring in clinical practice. We hypothesize that using cutoff values that clearly define pathology may improve the reliability of these scores. Therefore, the study aims to examine the validity of the OMERACT ultrasound score of KOA in contrast to clinical and radiographic data using predefined cutoff values in Egyptian patients and to assess the feasibility and inter-observer reliability between an experienced rheumatologist in musculoskeletal ultrasonography and a newly trained provider with no prior ultrasound experience.

By addressing these objectives, this study will contribute to a more comprehensive understanding of the OMERACT ultrasound knee OA scores’ validity, reliability, and feasibility in the Egyptian healthcare context, enhancing the global applicability of OMERACT—ultrasound scoring in knee OA management.

## Patients and methods

### Study design

This is a cross-sectional study that included 75 Egyptian patients with primary KOA who were recruited from the Outpatient Immunology Clinic and Inpatients Internal Medicine Department of Kasr Alainy Hospital. The inclusion criteria included patients aged more than 40 years and fulfilled the American College of Rheumatology (ACR) classification clinical and radiographic criteria for KOA [22]. Patients with a history of knee arthroplasty, systemic, or inflammatory joint disease; history of crystalline or neuropathic arthropathy; or those who received recent corticosteroid knee joint injections (< 6 weeks) were excluded. The study was approved by the Research Ethics Committee of the Faculty of Medicine, Cairo University. Informed written consent was taken from all patients.

### Clinical assessment

All patients were subjected to thorough history including age, medication profile, comorbid conditions, symptom duration, the duration of stiffness in the morning and after sitting, and the patient’s global assessment of knee pain using a 100 mm visual analogue scale (VAS) [23] and the Western Ontario and McMaster Universities Osteoarthritis Index (WOMAC). The WOMAC score is composed of 24 questions covering three dimensions: pain (five questions), stiffness (two questions), and function (17 questions) [24]. Clinical examination included body mass index (BMI), clinical assessment of knee joint effusion**,** knee deformities, and evaluation of range of movement (ROM) measured by goniometer. If patients had bilateral knee OA, the most symptomatic knee was selected.

### Radiographic assessment

Patients underwent bilateral weight-bearing fixed flexion posteroanterior knee radiography [25]. The radiography was graded using Kellgren and Lawrence [26] and the Osteoarthritis Research Society International (OARSI) grading system [27], by a physician who was blind to clinical and ultrasound assessments.

### Ultrasonography assessment

Knee ultrasonography was performed using a LOGIQ-P6 machine equipped with high-frequency broadband linear array transducer, at 10–13 MHz, within a week of clinical and radiographic assessments by one operator with more than 15 years of experience in musculoskeletal ultrasonography and was blind to the clinical and radiographic data. The machine settings of B-mode and power Doppler (PD) were adjusted to optimize knee image resolution and sensitivity to detect flow, respectively.

The following structures were evaluated according to the OMARACT knee ultrasound score [20]; however, some modifications were made including using cutoff values to determine the degrees of medial meniscus extrusion and osteophytes more accurately, we also used cutoff values to determine the presence of pathological effusion, and finally, we followed the updated OMERACT definition of synovitis:

1. The femoral cartilage was assessed while the patients were in the supine position, their knees were in possible maximum flexion, and the probe was placed transverse to the long axis of the femur just cranial to the patella. The cartilage damage was graded semi-quantitatively (0–3) as; 0: normal, Grade 1 (mild): irregularities or loss of sharpness of superficial and/or deep cartilage margins without thinning, Grade 2 (moderate): partial or complete loss of thickness of the cartilage in one trochlear facet, Grade 3 (marked): partial or complete loss of thickness of the cartilage in both trochlear facets.
2. Medial meniscal extrusion (MME): the extrusion of the anterior horn of the medial meniscus was assessed in the longitudinal scan of the medial tibio-femoral joint as protrusion outside the joint line. The probe was moved from anterior to posterior side and the maximum change was recorded. The distance of protrusion was measured and was graded semi-quantitatively (0–2) using previously determined cutoff values as follows: Grade 0 =  < 2 mm protrusion outside the femorotibial joint line, Grade 1 =  ≥ 2 mm and < 4 mm protrusion, Grade 2 =  ≥ 4 mm protrusion [15].
3. Osteophytes were assessed in the longitudinal scan of the medial joint space for medial osteophytes and of lateral joint space for lateral osteophytes. The probe was moved anterior to the posterior side and the maximal change was recorded, also both marginal bones were assessed and the one having the highest grade was recorded. Osteophytes were graded semi-quantitatively as follows: Grade 0 = no osteophytes, i.e., a smooth cortical surface; Grade 1 = small and distinct cortical protrusion(s) of the bony surface < 2 mm; Grade 2 = larger protrusion(s) of the bony surface with a size ranging from 2.1 to 4.0 mm; Grade 3 = very large protrusion(s) of the bony surface ≥ 4.1 mm [28].
4. Knee effusion is defined as abnormal hypoechoic or anechoic intraarticular material that is displaceable and compressible but does not exhibit Doppler signal. It was assessed longitudinally in the three knee recesses. The suprapatellar recess was evaluated while the knee flexed 30°. Both lateral and medial parapatellar recesses were assessed while the patient was in a neutral position. It was scored binary (0–1); it was considered present if the maximum anteroposterior measurement was ≥ 3.6 mm or ≥ 2.4 mm in suprapatellar or parapatellar recesses, respectively [7, 29].
5. Synovial hypertrophy is defined as abnormal hypoechoic, sometimes isoechoic, or hyperechoic intraarticular tissue that is partially displaceable and poorly compressible and which may exhibit a Doppler signal. It was assessed in the same scanned sites as effusion and by same planes. It also was scored binary (0–1).
6. Synovial power Doppler signal was scored binary (0–1). It was assessed in the same scanned sites as effusion and by the same planes.
7. Synovitis: is defined as the presence of a hypoechoic synovial hypertrophy regardless of the presence of effusion or any grade of Doppler signal [9]. It was scored semi-quantitively (0–3) as follows: Grade 0 = no synovitis; Grade 1 = minimal distension of the recess; Grade 2 = moderate distension or enlargement of the recess with flat or concave superficial limit; Grade 3 = severe distension or enlargement of the recess with bulging superficial limit. The three knee recesses were assessed and the one having the highest grade was recorded.

To evaluate the validity, the maximum knee score for each feature of the seven components was used to correlate with clinical and radiographic data. To test reliability, the machine settings were kept during the reliability exercise except for the position of the focus and the color box size/location according to the depth and size of the examined structure, respectively. The inter-observer reliability was evaluated in 30 knees by another newly trained operator who is expected to gain competency in performing the scans independently after a focused training period (12 h workload over 4 weeks). To assess feasibility, we considered this score to be feasible if it is easily done and consumes a short time; these two elements are important in real-time practice. This was evaluated by comparing the mean ultrasound examination time and reliability results between the two operators: the experienced and the newly trained one.

### Sample size

Sample size is calculated according to the following formula: total sample size = [(Zα + Zβ) / *C*]^2^ + 3; the standard normal deviate for *α* = Zα = 1.9600. The standard normal deviate for *β* = Zβ = 0.8416, *C* = 0.5 x [(1 + *r*) / (1 − *r*)] = 0.3428, where *r* = 0.33. The minimum sample size suitable for Pearson correlation of expected correlation coefficient (*r*) 0.33 is 70 [30].

### Statistics

The collected data were computerized and statistically analyzed using the SPSS program (Statistical Package for Social Science) version 27. Numerical data were described as mean and standard deviation or median and range. Categorical data were described as numbers and percentages and compared by Chi-square test. Data were explored for normality using the Kolmogorov–Smirnov test and the Shapiro–Wilk test. Comparisons between two groups for normally distributed variables were done by independent *T*-test and Mann–Whitney test for non-normally distributed numeric variables. The ANOVA test and its non-parametric analogue (Kruskal Wallis) were used to compare numerical variables between three groups or more followed by Bonferroni post hoc test and Dunn test, respectively. As a measure of reliability, Cohen’s Kappa was used to assess the degree of agreement between the newly trained operator and the experienced one. As a measure of reliability, Kappa should be greater than 0.70 (70%) not only to be significant, but Kappa more than 0.40 is to be considered fair. All statistical comparisons were two-tailed with a significance level of *p*-value ≤ 0.05 indicating significance and *p* 0.05 indicating a non-significant difference.

## Results

### Demographic and clinical characteristics of patients

Seventy-five patients were included in this study; their characteristics are illustrated in Table 1. The majority of our patients were female (78.7%), their ages ranged from 48 to 85 years, and their mean BMI reflects obesity (33.9 ± 6.8 kg/m^2^). The patients had high scores of total WOMAC, and the mean of their knee pain using 100-VAS reflects severe pain (60 ± 20). All included patients had symptoms in both knees. Table 1 presents the characteristics of the most affected knee. Seventeen (23%) patients had clinically detected knee effusion, and 41 (55%) patients had knee deformities in the form of knock-knees or bowlegs; also, they had limited range of movement (ROM).

**Table 1:** Patients’ demographic, clinical and radiographic characteristics of the most symptomatic knee

Number	75 patients
Age (years) (mean ± SD)	63.5 ± 8.9
Sex: male/female (number (%))	16 (21.3)/59 (78.7)
Comorbid conditions (number (%))	42 (56.0)
Diabetes (number (%))	33 (79)
COPD (number (%))	2 (5)
Hypertension (number (%))	27 (64)
Current medication profile (number (%))	75 (100)
Topical treatments (Caspian/NSAIDs) (number (%))	60 (80)
Analgesics (number (%))	30 (40)
Supplementary drugs (such as glucosamine/chondroitin sulfate/others) (number (%))	60 (80)
Weight (kg) (mean ± SD)	84 ± 17.5
BMI (kg/m^2^) (mean ± SD)	33.9 ± 6.8
Duration of symptoms (years) (median (IQR))	2 (4)
Duration of morning stiffness (minutes) (mean ± SD)	13 ± 2
Knee pain using 100-VAS (mean ± SD)	60 ± 20
Knee deformities (number (%))	41 (55)
Clinically detected knee effusion (number (%))	17 (23)
Extension degree (mean ± SD)	163 ± 12
Flexion degree (mean ± SD)	58 ± 13
WOMAC score (mean ± SD)	
Total WOMAC	53 ± 17
WOMAC–pain score	9 ± 5
WOMAC–stiffness score	4 ± 2
WOMAC–physical score	40 ± 19
Kellgren Lawrence grade (KLG) [frequency (%)]	
Grade 1	11 (15)
Grade 2	35 (47)
Grade 3	22 (29)
Grade 4	7 (9)

*SD* standard deviation, *COPD* chronic obstructive pulmonary disease, *BMI* body mass index. Comorbid conditions such as diabetes and hypertension. *WOMAC* Western Ontario and McMaster Universities Osteoarthritis Index, *SD* standard deviation, *VAS* visual analogue scale, *KLG* Kellgren Lawrence grade

### Radiographic characteristics

Table 1 demonstrates the Kellgren Lawrence grade (KLG) of the most symptomatic knees (Table 1). Sixty-four (85%) knees had radiological KOA defined as KLG ≥ 2.

### Ultrasonography characteristics

Ultrasound features of the most symptomatic knee are detailed in Table 2. High grades of structural changes were found in the majority of enrolled patients. On the other hand, while effusion was found in 67 patients (89%), synovitis and synovial hypertrophy were only found in 15 patients (20%). Synovial power Doppler was found in only one patient. Representative ultrasound images are shown in (Supplementary Fig. 1).

**Table 2:** Ultrasound findings of the most symptomatic knee

	Frequency (%)
**Femoral cartilage damage**	
Normal	1 (1)
Mild	9 (12)
Moderate	37 (49)
Marked	28 (37)
**Medial meniscus extrusion (MME) grade**	
Grade 0 (< 2 mm) (normal)	1 (1)
Grade1 (≥ 2 mm– < 4 mm)	14 (19)
Grade 2 (≥ 4 mm)	60 (80)
**Medial osteophytes**	
No osteophytes	5 (7)
Small osteophytes	6 (8)
Large protruded osteophyte	21 (28)
Very protruded osteophyte	43 (57)
**Lateral osteophytes**	
No osteophytes	18 (24)
Small osteophyte	11 (15)
Large protruded osteophyte	25 (33)
Very protruded osteophyte	21 (28)
**Effusion**	67 (89)
**Synovial hypertrophy**	15 (20)
**Backer cyst**	15 (20)
**Synovitis**	
Grade 2	9 (12)
Grade 3	6 (8)

### Associations between ultrasound features and WOMAC score

The presence of synovitis was the only US finding that had a statistically significant association with WOMAC–pain score (*p*-value 0.046). Grade 2 MME and large protruded medial osteophytes were associated with increased pain sub-score, however not statistically significant. MME and medial osteophytes were significantly associated with stiffness sub-score (*p*-value 0.009 and 0.023, respectively). MME and synovitis were significantly associated with WOMAC–physical sub-score (*p*-value 0.035 and 0.020, respectively). Marked femoral cartilage damage was associated with a higher WOMAC–physical sub-score, however not statistically significant (Table 3).

**Table 3:** Associations between US features and WOMAC scores

Ultrasound features	Pain score		Stiffness score		Physical score	
	Median	*p*-value	Median	*p*-value	Median	*p*-value
**Femoral cartilage damage**						
Mild	12	0.275	4	0.724	22	0.531
Moderate	10		4		45	
Marked	9		4		52	
**Medial meniscus extrusion**						
Grade 1	7	0.121	2	0.009*	60	0.035*
Grade 2	10		4		39	
**Effusion**	10	0.897	4	0.375	42	0.358
**Medial osteophytes**						
No osteophytes	7	0.197	3	0.023*	60	0.066
Small osteophyte	6		2		60	
Large protruded	11		4		37	
Very protruded	9		4		42	
**Lateral osteophytes**						
No osteophytes	6	0.097	4	0.080	60	0.266
Small osteophyte	11		4		47	
Large protruded	10		4		37	
Very protruded	10		4		35	
**Synovitis**	11	0.046	4	0.614	34	0.020*

* Statistically significant *p*-value

### Associations between ultrasound features and 100-VAS scale of knee pain

The presence of synovitis and synovial hypertrophy was significantly associated with pain severity using 100-VAS (*p*-value 0.021 and 0.028, respectively). The presence of effusion alone was not associated with pain severity (Supplementary Table 1).

### Associations between ultrasound features and radiographic OARSI score

A total of 150 knees of 75 patients were included in the analysis. The ultrasonographic femoral cartilage damage and MME that reflects joint narrowing were significantly associated with OARSI narrowing of medial tibiofemoral joint space (*p*-value 0.041 and < 0.001, respectively) (Table 4). The ultrasonographic detected medial and lateral osteophytes were significantly associated with OARSI medial and lateral osteophytes (*p*-value 0.014 and < 0.001, respectively) (Table 5).

**Table 4:** Association between US features and OARSI score for medial tibiofemoral joint space narrowing for all knees (*n* = 150 knees)

OARSI score for medial tibiofemoral joint space narrowing	Ultrasound detected femoral cartilage damage			Ultrasound detected medial meniscus extrusion		
	Mean ± SD	Median (range)	*p*-value	Mean ± SD	Median (range)	*p*-value
**No narrowing**	1.9 ± 0.7	2 (1–3)	0.041*	1.9 ± 0.5	1.9 (1.3–2.8)	< 0.001*
**Mild**	1.9 ± 0.8	2 (1–3)		4.2 ± 2.2	3.5 (1.6–11.6)	
**Moderate**	2.2 ± 0.7	2 (0–3)		6.1 ± 2.4	6.3 (0.9–12.1)	
**Severe**	2.4 ± 0.6	2 (1–3)		9.2 ± 4.2	9.1 (1–25.2)	

*OARSI* Osteoarthritis Research Society International Score, * statistically significant *p-value*

**Table 5:** Associations between the ultrasound score of osteophytes and OARSI score of osteophytes for all knees (*n* = 150 knees)

		OARSI score of medial osteophytes (frequency (%))				Kappa	*p*-value
		No	Small	Large protruded	Very protruded		
**Ultrasound score of medial osteophytes** (frequency (%))	**No**	7 (4.7)	5 (3.3)	0 (0)	0 (0)	0.098	0.014*
	**Small**	13 (8.7)	7 (4.7)	1 (0.7)	0 (.0)		
	**Large protruded**	10 (6.7)	21 (14.0)	10 (6.7)	3 (2.0)		
	**Very protruded**	6 (4.0)	21 (14.0)	26 (17.3)	20 (13.3)		
**Ultrasound score of lateral osteophytes** (frequency (%))	**No**	32 (21.3)	3 (2.0)	3 (2.0)	0 (0)	0.150	< 0.001*
	**Small**	17 (11.3)	9 (6.0)	1 (0.7)	0 (0)		
	**Large protruded**	9 (6.0)	23 (15.3)	10 (6.7)	2 (1.3)		
	**Very protruded**	5 (3.3)	14 (9.3)	20 (13.3)	2 (1.3)		

* Statistically significant *p*-value

### Inter-observer reliability of OMERACT-US score

Thirty patients were assessed in the reliability exercise. The kappa statistics for inter-observer reliability ranged from 0.58 to 0.83 indicating moderate to excellent agreement. The reliability was highest for the assessment of lateral osteophytes (*k* = 0.83) with excellent agreement, followed by good agreement for synovitis (*k* = 0.72), both osteophytes (*k* = 0.71), and damage of femoral cartilage (*k* = 0.70). The reliability was least for the assessment of medial osteophytes (*k* = 0.58) and MME (*k* = 0.59) with moderate agreement (Table 6).

**Table 6:** Inter-observer reliability

Ultrasound score	Observer one	Observer two				Measurement of agreement	
		0	1	2	3	Kappa	*p*-value
**Synovitis**	**0**	7	0	0	0	**0.724**	0.000*
	**1**	0	2	3	0		
	**2**	1	1	8	0		
	**3**	0	0	1	7		
**Damage of femoral cartilage**	**0**	1	1	0	0	**0.703**	0.000*
	**1**	0	4	0	0		
	**2**	0	2	10	2		
	**3**	0	0	1	9		
**Medial osteophytes**	**0**	2	0	1	0	**0.582**	0.000*
	**1**	2	4	2	0		
	**2**	0	1	8	1		
	**3**	0	0	2	7		
**Lateral osteophytes**	**0**	8	0	0	0	**0.838**	0.000*
	**1**	1	3	0	0		
	**2**	0	0	15	1		
	**3**	0	0	1	1		
**Both osteophytes**	**0**	10	0	1	0	**0.712**	0.000*
	**1**	3	7	2	0		
	**2**	0	1	23	2		
	**3**	0	0	3	8		
**Medial meniscal extrusion**	**0**	1	0	0		**0.596**	0.000*
	**1**	2	5	4			
	**2**	0	0	18			

* Statistically significant *p*-value

### Feasibility time

Using the standardized protocol, the time taken by the experienced operator (Examiner-1) to obtain high-quality images for scoring was significantly shorter compared to the time taken by the newly trained operator (Examiner-2) (mean time ± SD of 5.36 ± 0.8 min versus 5.44 ± 0.9 min per knee, *p*-value 0.001]. Also, Examiner-1 took a shorter duration to interpret the US images than Examiner-2 (mean time ± SD of 3.7 ± 0.48 versus 5.2 ± 0.16 min per knee, *p*-value 0.002). Accordingly, the mean total time taken by the experienced operator was 9.09 ± 0.5 min, which was significantly less than the time taken by the newly trained operator, which was 10.66 ± 0.2 min (*p*-value < 0.001) (Supplementary Table 2).

## Discussion

In this study, we evaluated the applicability of the OMERACT-US score of KOA in 75 Egyptian patients with primary KOA, by using the three filters previously proposed by OMERACT to endorse a measure: validity, reliability, and feasibility [31].

Compared with the standard score, we used the cutoff values defined in the literature to grade MME and osteophytes. We also believed it was important to distinguish between pathological and physiological knee fluid that should not be scored. We preferred to use sensitive cutoff values that have been proposed for abnormal fluid in the knee recesses, which are 2.4 mm for both parapatellar recesses in the neutral position and 3.6 mm for the midline of the suprapatellar recess when the knee is flexed to 30°. It should be noted that a cutoff value of 1.8 mm increases the sensitivity for the lateral parapatellar recess; however, we preferred to standardize the measurement between the two parapatellar recesses [29]. Another important modification was that we followed the updated OMERACT definitions in which synovial hypertrophy is mandatory for defining the presence of an US-detected synovitis [9]. For this reason, although effusion was recorded in 89% of our patients, synovitis was graded in only 20% of them in whom synovial hypertrophy was detected.

### Association between OMERACT-US score and clinical outcomes

The study found that higher OMERACT-US synovitis scores were significantly associated with greater pain levels and worse physical function, as measured by the respective sub-scores of the WOMAC, and higher ratings of pain intensity on the 100-point VAS. The binary score of synovial hypertrophy, the essential component of synovitis, was only significantly associated with the 100-VAS pain intensity rating. This questioned the added value of including a binary score for synovial hypertrophy.

These findings align with several previous studies that have demonstrated that ultrasound-detected synovitis was significantly associated with greater pain intensity using the VAS [32] as well as worse scores on other clinical outcomes such as Knee Injury and Osteoarthritis Outcome Score (KOOS) [21, 19] and the Intermittent and Constant Osteoarthritis Pain (ICOAP) tool [33] when compared to patients without evidence of synovitis.

In our study, effusion was prevalent among the patients; however, the OMERACT-US score of effusion was not associated with total WOMAC and its sub-scores or pain intensity on the VAS. We assumed that the binary score of effusion might have an underestimated association with clinical outcomes that would be detected if semi-quantitative graded scores were used. This assumption is supported by the findings from Philpott et al., who reported that for every 1 mm increase in the maximum depth of effusion, there was an increase of 2.04 (95% CI 0.89–3.20) points in constant pain score [33]. Also, Yerich et al. found that cases with moderate to severe effusion were more likely to report higher pain levels and diminished functioning [19].

Regarding the structural components of the OMERACT score, including MME, osteophytes, and femoral cartilage damage, the current study found notable associations with WOMAC sub-scores. The MME score was significantly associated with higher levels of stiffness and worse physical function based on respective WOMAC sub-scores. The MME score of Grade 2, representing ≥ 4 mm of extrusion based on the cutoff used, was associated with a higher WOMAC pain sub-score, although this association did not reach statistical significance. The osteophyte score was associated significantly with the WOMAC stiffness sub-score. Large protruded medial osteophytes were associated with increased WOMAC pain sub-score, but also this association was not statistically significant. Finally, the femoral cartilage damage score only exhibited a positive association with worse function as measured by the WOMAC physical function sub-score, but this association was not statistically significant. Studies have shown conflicting results regarding the clinical implications of ultrasound-detected MME. Some studies have shown that MME was significantly associated with higher levels of pain measured by different tools like the Numerical Pain Rating Scale (NRS), KOOS pain score, other KOOS symptoms [21], and WOMAC scores [34], and MME has also been associated with pain when climbing stairs [35]. However, other studies have not found a significant relationship between MME and pain. Yerich et al. found that while meniscal extrusion increased the odds of KOOS pain by around 20%, this increase was not statistically significant [19]. Also, Wu et al. did not find any association between MME and pain measured by the VAS during movement, VAS at rest, or the WOMAC pain sub-score [32].

To potentially resolve the inconsistent findings regarding the relationship between meniscal extrusion and clinical outcomes, we hypothesized that using a cutoff value to grade meniscal extrusion could be helpful. Kijima et al. reported that meniscal extrusion greater than 4.3 mm was associated with knee pain with a sensitivity of 0.8451 and specificity of 0.8502 [36]. Additionally, Ishii et al. divided patients with KOA into two groups based on the extent of the severity of MME: those with MME ≥ 3 mm were categorized as severe, while those with MME < 3 mm were considered mild; they found that patients with an MME ≥ 3 mm under weight-bearing conditions experienced more severe knee pain, as assessed by the KOOS pain score, compared to those with less extruded meniscus (< 3 mm) [37]. These findings suggest that establishing specific cutoff values for grading MME severity could help clarify its relationship with knee pain levels.

Regarding the OMERACT-US scores for osteophyte and femoral cartilage damage, our findings align with those of Oo et al. Their study demonstrated a significant association between the OMERACT-US score of medial osteophytes and KOOS other symptoms subscale, but not the KOOS pain subscale. Additionally, they found that the OMERACT-US score of femoral cartilage damage did not have any significant associations with either the KOOS other symptoms or KOOS pain subscales [21].

### Association between OMERACT-US score and radiographic features

The current study showed that the OMERACT-US scores for femoral cartilage damage, MME, and osteophytes had significant associations with the corresponding OARSI radiographic scores. Notably, MME demonstrated a more significant association with joint space narrowing scored by the OARSI than with femoral cartilage damage. We used the OARSI radiographic score to correlate with ultrasound findings, as this score provides separate scores for tibiofemoral joint space narrowing and osteophytes, allowing us to compare X-ray scores with similar ultrasound features. This may explain why other studies have found significant associations between ultrasound-detected osteophytes and MME scores with KLG. But there is no significant association between ultrasound-detected cartilage damage and KLG [21, ]. The KLG is a global score and may not be ideal for validating individual components assessed by ultrasound.

### Reliability of OMERACT ultrasound score

Ultrasound is an operator-dependent technique. We then examined the interobserver reliability between an experienced and a new trainee in musculoskeletal ultrasound; the results showed excellent reliability for assessing lateral osteophytes, good reliability for synovitis and femoral cartilage damage, and moderate reliability for evaluating medial osteophytes and MME. Regarding the synovial PD signal, its low prevalence in our sample prevented us from calculating the reliability of this parameter.

When comparing our results to those reported in the original OMERACT reliability study [20], we found comparable reliability for MME (*k* = 0.59 vs 0.56). However, our results showed higher interobserver agreement for synovitis (*k* = 0.72 vs 0.50), osteophytes (*k* = 0.71 vs 0.60), and cartilage damage (*k* = 0.70 vs 0.34) compared to the OMERACT study. It is important to note that the original OMERACT study included only well-experienced sonographers [20], whereas our study compared two operators with different levels of experience. We assumed that using specific cutoff values to score MME and osteophytes, in addition to a focused training period to gain competency in performing the ultrasound scan and interpretation, is a key factor that may contribute to improving reliability results.

In a systematic literature review and meta-analysis, pooled kappa values for the interrater reliability of the semi-quantitative scores were moderate for cartilage thickness (0.44 (0.15–0.74)) and substantial for MME (0.75 (0.41, 1)), synovitis (0.63 (0.36, 0.90)), and osteophytes (0.66 (0.50, 0.82)) [10].

### Feasibility of OMERACT ultrasound score

In our study, the experienced operator completed the protocol for obtaining high-quality knee images and interpreting them in a shorter time compared to the newly trained operator. Nevertheless, both operators’ completion times were considered short, supporting the feasibility of using the OMERACT-US score in clinical practice.

This time frame aligns with Bruyn et al., who also used the OMERACT–US score, where each sonographer took a maximum of 8 min to scan both knees and completed a standardized report sheet with the US findings [20]. Oo et al. reported a longer time using the same score: approximately 8 min for scanning the entire knee and about 13 min for scoring [21]. Yerich et al. employing a different ultrasound score took 10–15 min for scanning and 3–5 min for image interpretation [19]. A systematic literature review and meta-analysis of five studies reported that the scanning time for a complete examination ranged from 5 to 15 min, depending on the number of pathologies evaluated [10].

We believe that this research on the OMERACT ultrasound scoring system for KOA has significant practical implications across various sectors. For society and healthcare systems, ultrasound is generally more accessible and cost-effective than other imaging modalities like MRI. The validation of this scoring system could lead to more widespread use of ultrasound in KOA assessment, potentially reducing healthcare costs associated with more expensive imaging techniques. Healthcare professionals benefit from a standardized, reliable tool that complements clinical assessments and could lead to more personalized treatment plans and better monitoring of disease progression. Researchers gain a validated method for quantifying both structural and inflammatory aspects of KOA, enhancing the comparability of clinical trials. Overall, this advancement represents a significant step towards a more accurate, consistent, and comprehensive assessment of KOA, with the potential to improve patient care and our understanding of this common condition.

## Limitations

Our study had limitations. Firstly, a significant proportion of our patients were obese (BMI > 30), and 55% had knee deformities. This made accurate assessment of femoral cartilage challenging, as it requires full knee flexion. Secondly, while radiographic images were obtained in a weight-bearing position, the ultrasound examination was performed with patients lying supine. It is difficult for osteoarthritis patients to stand during an ultrasound examination. Lastly, the cross-sectional nature of our study only allowed us to describe associations at a single time point. Longitudinal studies would be preferable to assess the causal relationships between ultrasound features and clinical as well as radiographic outcomes.

## Conclusion

In conclusion, our study validates the use of OMERACT-US scores for KOA in Egyptian patients, with scores showing significant associations with WOMAC clinical scores. However, the binary score for effusion did not reflect clinical outcomes, suggesting that a semi-quantitative grading system may be necessary to capture the impact of effusion on clinical outcomes more accurately. The binary score for synovial hypertrophy can be omitted as it provided no added value. Furthermore, the OMERACT-US scores demonstrated strong associations with the radiographic OARSI scores.

Our study showed that inter-observer reliability ranged from moderate to excellent agreement, indicating that OMERACT ultrasound knee osteoarthritis scores are reliable, particularly when cutoff values are included, and proper training time is provided. The ultrasound examination was completed in a short time, even by a newly trained operator, demonstrating the feasibility of using the OMERACT ultrasound knee osteoarthritis scores in clinical practice.

These findings support the potential for widespread adoption of this scoring system in both clinical and research settings for evaluating knee osteoarthritis.

## Supplementary Information

Below is the link to the electronic supplementary material.

Supplementary file1 (PDF 660 KB)

Supplementary file2 (PDF 135 KB)
