# Supplementary material for: Assessment of validity, reliability, and feasibility of OMERACT ultrasound knee osteoarthritis scores in Egyptian patients with primary knee osteoarthritis
Source: Clin Rheumatol. 2024 Oct 18;43(12):3913–23. doi: 10.1007/s10067-024-07171-4 (PMC11582230; doi:10.1007/s10067-024-07171-4)
Supplement: Supplementary file 1 — Supplementary file1 (PDF 660 KB) [file 10067_2024_7171_MOESM1_ESM.pdf]

Journal name	Clinical Rheumatology
Complete manuscript title	***Assessment of Validity, Reliability and Feasibility of OMERACT Ultrasound Knee Osteoarthritis Scores in Egyptian Patients with Primary Knee Osteoarthritis***
Authors	**Manal Abd El Moniem El Menyawi [1a], Galila Gamal [2a], Hoda Abdelbadie [3b] , Rasmia Elgohary[3a].**
Authors affiliations	[1] Professor, [2] Assistant lecturer, [3] Assistant professor.<br>[a] Rheumatology and Clinical Immunology Subspeciality, Internal Medicine Department, Kasr Alainy School of Medicine, Cairo University, Egypt<br>[b] Rheumatology and Clinical Immunology Subspeciality, Internal Medicine Department, Fayoum University, Egypt
Corresponding author	Rasmia Elgohary<br>• Address: Cairo University Hospitals, Al-Saray St., El-Maniel, 11562, Cairo, Egypt. Email:<br>• rasmiaelgohary@kasralainy.edu.eg<br>• Tel.: +201111370118<br>• ORCID: 0000-0002-4002-1485

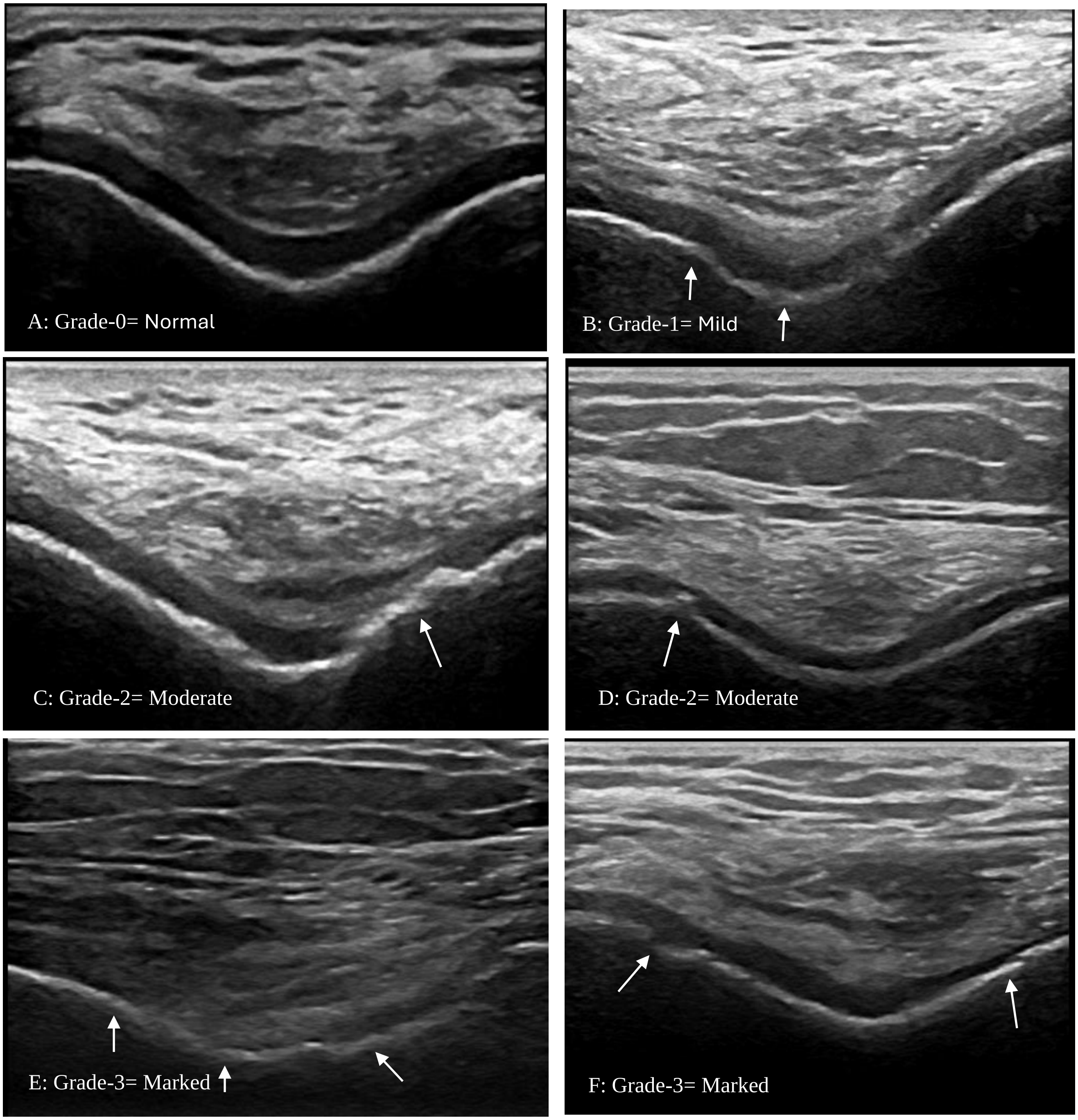

Figuer-1: The 0-3 semi-quantitative grading of cartilage damage; 0: normal (A), Grade-1= mild (B): irregularities (arrows) or loss of sharpness of superficial and/or deep cartilage margins without thinning (arrow head), Grade-2= moderate: complete (C) or partial (D) loss of thickness of the cartilage in one trochlear facet, Grade 3 (marked): complete (E) or partial (F) loss of thickness of the cartilage in both trochlear facets.

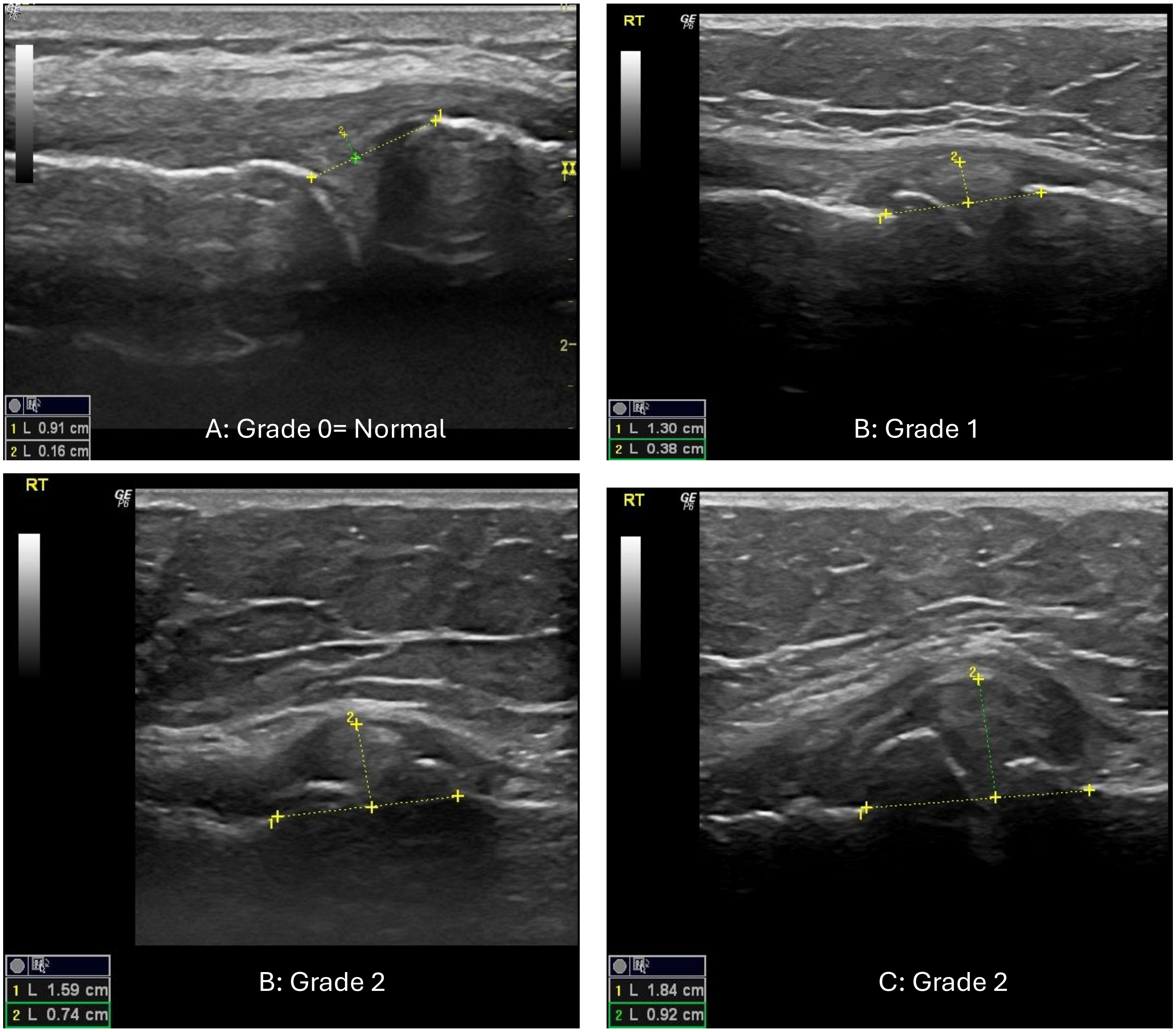

Figure 2: the 0-2 semi-quantitative grading of medial meniscal extrusion (MME); Grade 0= < 2 mm protrusion outside the femorotibial joint line (A), Grade 1= ≥ 2 mm and < 4 mm protrusion (B), Grade 2= ≥ 4 mm protrusion (C and D).

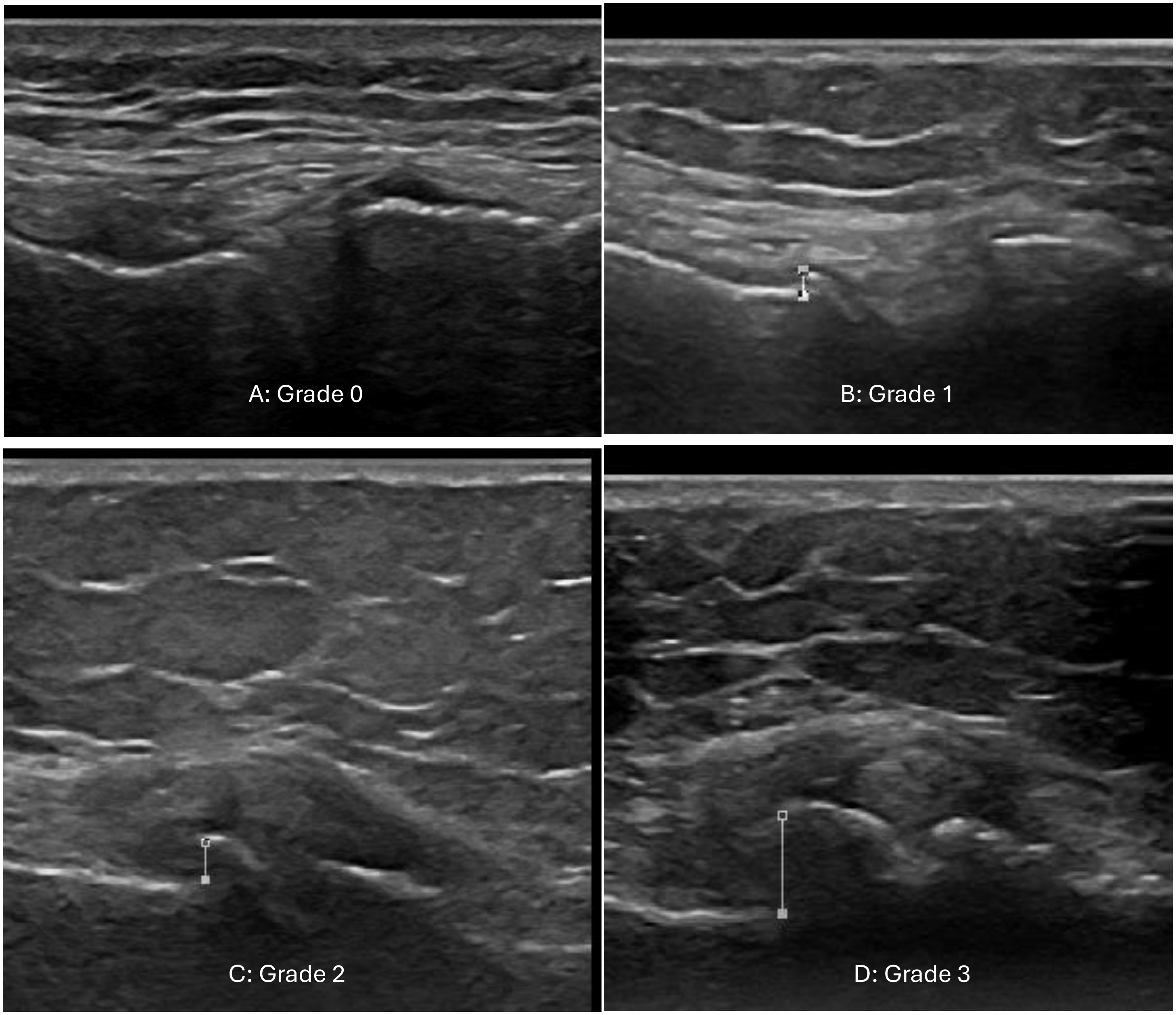

Figure 3: the semiquantitative grading of medial osteophytes: (A) Grade 0= no osteophytes. (B) Grade 1= small and distinct cortical protrusion(s) of the bony surface<2mm. (C) Grade 2= larger protrusion(s) of the bony surface with a size ranging from 2.1 to 4.0 mm. (D) Grade 3= very large protrusion(s) of the bony surface≥ 4.1 mm.

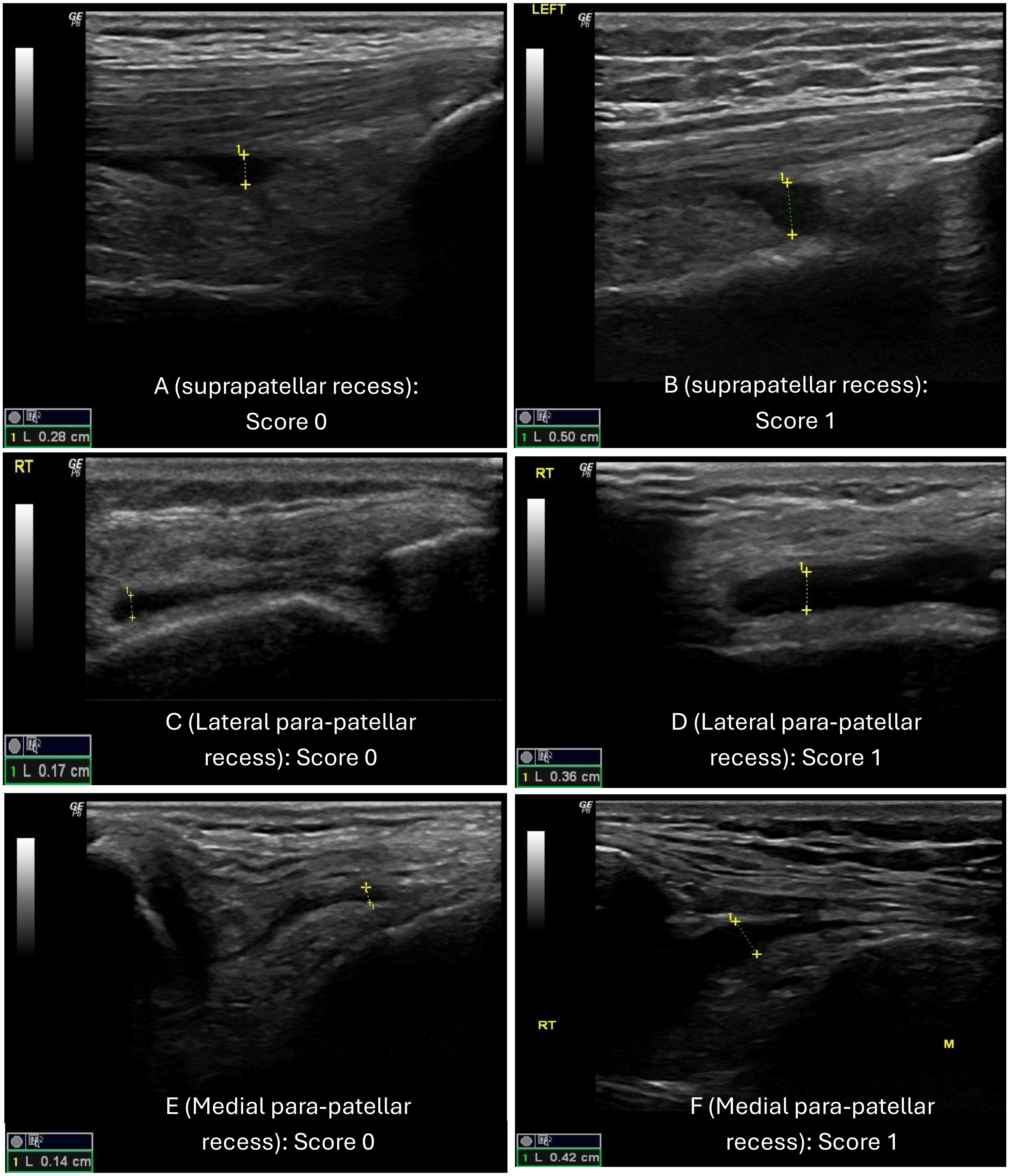

Figure 4: Binary score of effusion in the three knee recesses; Score 0= maximum anteroposterior measurement of effusion < 4 mm in suprapatellar recess (A), and < 2.4 mm in lateral (C) & medial (E) para-patellar recesses. Score 1= maximum anteroposterior measurement of effusion ≥ 3.6 mm in suprapatellar recess (B), and ≥ 2.4 mm in lateral (D) & medial (F) para-patellar recesses.

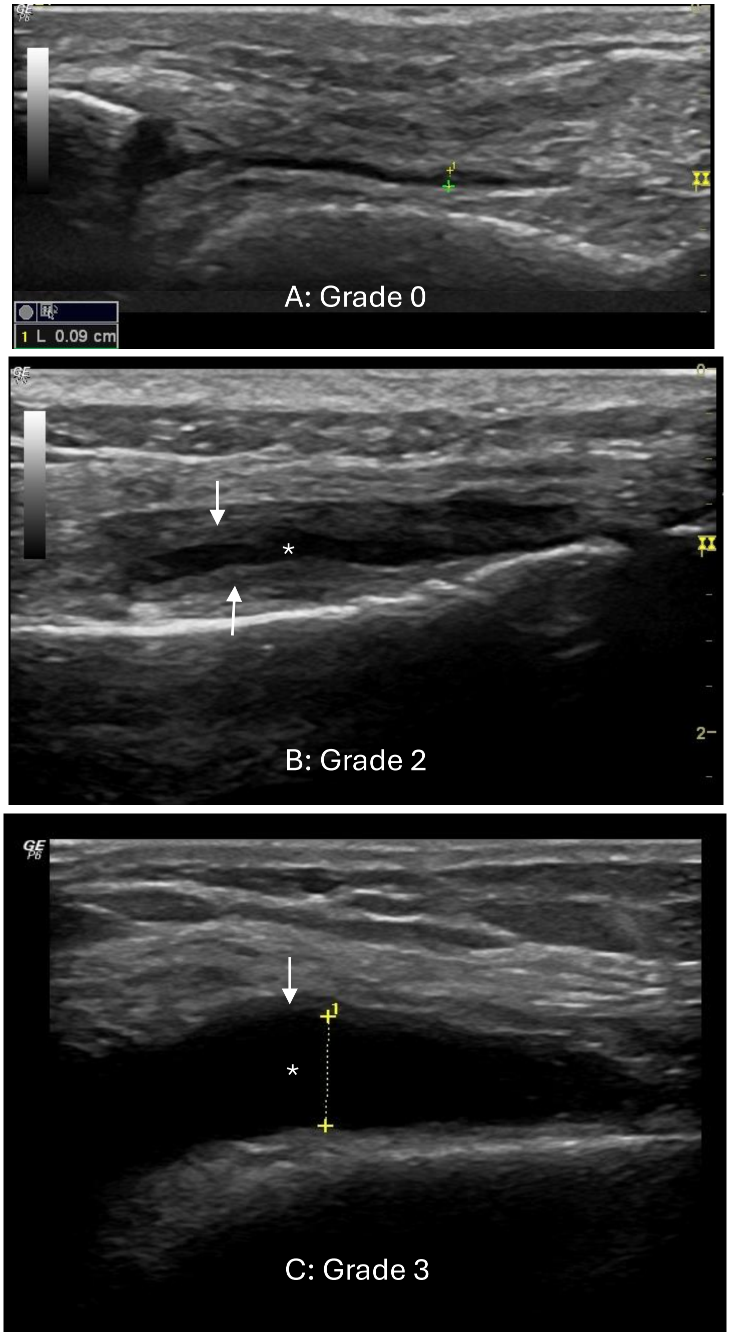

Figuer-5: Semi-quantitative grading of synovitis; (A) Grade 0: normal, (B) Grade-2: moderate distension or enlargement of the recess by synovial hypertrophy (arrows) and effusion (asterixis), (C) Grade-3: severe distension or enlargement of the recess by synovial hypertrophy (arrows) and effusion (asterixis). Grade 1 that represents minimal distension of the recess by synovial hypertrophy with or without effusion is not represented.
