# Supplementary material for: Assessment of validity, reliability, and feasibility of OMERACT ultrasound knee osteoarthritis scores in Egyptian patients with primary knee osteoarthritis
Source: Clin Rheumatol. 2024 Oct 18;43(12):3913–23. doi: 10.1007/s10067-024-07171-4 (PMC11582230; doi:10.1007/s10067-024-07171-4)
Supplement: Supplementary file 2 — Supplementary file2 (PDF 135 KB) [file 10067_2024_7171_MOESM2_ESM.pdf]

Journal name	Clinical Rheumatology
Complete manuscript title	***Assessment of Validity, Reliability and Feasibility of OMERACT Ultrasound Knee Osteoarthritis Scores in Egyptian Patients with Primary Knee Osteoarthritis***
Authors	**Manal Abd El Moniem El Menyawi [1a], Galila Gamal [2a], Hoda Abdelbadie [3b] , Rasmia Elgohary[3a].**
Authors affiliations	[1] Professor, [2] Assistant lecturer, [3] Assistant professor.<br>[a] Rheumatology and Clinical Immunology Subspeciality, Internal Medicine Department, Kasr Alainy School of Medicine, Cairo University, Egypt<br>[b] Rheumatology and Clinical Immunology Subspeciality, Internal Medicine Department, Fayoum University, Egypt
Corresponding author	Rasmia Elgohary<br>• Address: Cairo University Hospitals, Al-Saray St., El-Maniel, 11562, Cairo, Egypt. Email:<br>• rasmiaelgohary@kasralainy.edu.eg<br>• Tel.: +201111370118<br>• ORCID: 0000-0002-4002-1485

**Supplementary table 1: Association between US inflammatory features and VAS of knee pain.**		
	**knee pain using VAS [mean (SD)]**	**p value**
**Effusion**	59 (20)	0.952
**Synovial hypertrophy**	69 (16)	**0.028**
**Synovitis**	69 (15)	**0.021**

Supplementary table 2: Assessment of feasibility of ultrasound in knee osteoarthritis					
	Examiner 1		Examiner 2		p value
	Mean ±SD	Range	Mean ±SD	Range	
Examination time (minutes)	5.36±0.8	5.22 - 5.46	5.44±0.9	5.3-5.6	<0.001
Interpretation time (minutes)	3.7±0.48	3.2 - 4.5	5.2±0.16	5-5.6	0.002
Total time	9.09±0.5	8.42-9.96	10.66±0.2	10.3-11.2	<0.001
SD: standard deviation.					
